# Association between Blood Lead Levels and Delta-Aminolevulinic Acid Dehydratase in Pregnant Women

**DOI:** 10.3390/ijerph14040432

**Published:** 2017-04-18

**Authors:** Osmel La-Llave-León, Edna M. Méndez-Hernández, Francisco X. Castellanos-Juárez, Eloísa Esquivel-Rodríguez, Fernando Vázquez-Alaniz, Ada Sandoval-Carrillo, Gonzalo García-Vargas, Jaime Duarte-Sustaita, Jorge L. Candelas-Rangel, José M. Salas-Pacheco

**Affiliations:** 1 Institute of Scientific Research, Juarez University of the State of Durango, AV. Universidad y Fanny Anitua s/n. Col. Centro, C. P. 34000 Durango, Mexico; ollave56@yahoo.es (O.L.-L.L.); edna_madai@hotmail.com (E.M.M.-H.); xavier_castellanos@hotmail.com (F.X.C.-J.); adda-sandoval@hotmail.com (A.S.-C.); 2 Faculty of Nursing and Midwifery, Juarez University of the State of Durango, Cuauhtemoc, 223 North, Col. Centro, C. P. 34000 Durango, Mexico; eloesqui@yahoo.com.mx; 3 General Hospital 450, Health Services, C. P. 34000 Durango, Mexico; feralaniz1@hotmail.com; 4 Faculty of Health Sciences, Juarez University of the State of Durango, Gomez Palacio la Salle 1 y Sixto Ugalde, s/n, Col. Revolucion, C. P. 35050 Gomez Palacio, Durango, Mexico; ggarcia_vargas@hotmail.com (G.G.-V.); qfb.jaimeduarte@gmail.com (J.D.-S.); Jorge_candelas@hotmail.com (J.L.C.-R.)

**Keywords:** blood lead levels, delta-aminolevulinic acid dehydratase (ALAD) activity, pregnant women, lead exposure, lead toxicity

## Abstract

Blood lead levels (BLLs) and delta-aminolevulinic acid dehydratase (ALAD) activity are considered biomarkers of lead exposure and lead toxicity, respectively. The present study was designed to investigate the association between BLLs and ALAD activity in pregnant women from Durango, Mexico. A total of 633 pregnant women aged 13–43 years participated in this study. Blood lead was measured by a graphite furnace atomic absorption spectrometer. ALAD activity was measured spectrophotometrically. Mean blood lead was 2.09 ± 2.34 µg/dL; and 26 women (4.1%) crossed the Centers for Disease Control (CDC) recommended level of 5 µg/dL. ALAD activity was significantly lower in women with levels of lead ≥5 µg/dL compared to those with BLLs < 5 µg/dL (*p* = 0.002). To reduce the influence of extreme values on the statistical analysis, BLLs were analyzed by quartiles. A significant negative correlation between blood lead and ALAD activity was observed in the fourth quartile of BLLs (r = −0.113; *p* < 0.01). Among women with blood lead concentrations ≥2.2 µg/dL ALAD activity was negatively correlated with BLLs (r = −0.413; *p* < 0.01). Multiple linear regression demonstrated that inhibition of ALAD in pregnant women may occur at levels of lead in blood above 2.2 µg/dL.

## 1. Introduction

Lead is known to represent a significant environmental hazard to pregnant women and their offspring. Exposure to high environmental levels of lead during pregnancy has been associated with some adverse outcomes [1]. However, recent findings indicate that lead may be toxic at levels previously considered to have no adverse effects. Research suggests that lead exposure at both low and high concentrations adversely affects hematopoietic, vascular, nervous, renal and reproductive systems [2]. During pregnancy, adverse reproductive outcomes may occur at levels of lead in blood below 10 μg/dL. Infertility [3], spontaneous abortion [4], preeclampsia [5,6,7] and preterm delivery [8] have all been associated with lead exposure at levels previously considered safe.

Blood lead concentrations above 2.5 μg/dL have been associated with an increased risk of infertility [3]. A significant association between blood lead concentrations and hypertension during pregnancy has been documented [5,7]. Significantly higher blood lead levels have been reported in women with pregnancy-induced hypertension compared to normotensive patients, and significant correlations between blood lead levels and systolic and diastolic blood pressures have been found [7]. Moreover, higher levels of lead in umbilical cord blood have been found in preeclampsia cases compared to women without this condition [5].

Elevated lead levels have been also associated with abortion and duration of pregnancy [4,8]. In a prospective study in Mexico city a statistically significant relationship between low-to-moderate maternal lead levels and the risk of spontaneous abortion was demonstrated [4]. Furthermore, researchers have found significantly higher blood lead levels during the first trimester of pregnancy in mothers who delivered preterm babies when compared with those whohadfull-term pregnancies [8].

Several biological techniques and biomarkers are useful for risk assessment of lead in the field of environmental health. Blood lead is the most widely used biomarker of lead exposure. This indicator represents a measure of soft tissue lead, body burden and absorbed doses of lead, whereas the critical effects of lead in bone marrow can be used as biomarker of effect. The effects of lead in bone marrow arise mainly from lead interaction with some enzymatic processes involved in heme synthesis [9]. 

The main biomarkers of effect are the inhibition of delta-aminolevulinic acid dehydratase (ALAD) and the variation in some metabolite concentrations, such as zinc protoporphyrin (ZP) in blood, delta-aminolevulinic acid in urine (ALA-U), delta-aminolevulinic acid in blood (ALA-B), delta-aminolevulinic acid in plasma (ALA-P) and coproporphyrin in urine (CP). However, not all mentioned indicators equally reflect dose and internal dose/effect relationship [2].

Lead toxicity may be explained by its interference with different enzymes. Lead inactivates these enzymes by binding to the SH-groups of proteins or by displacing some essential metal ions. Lead is known to inhibit three enzymes involved in the heme pathway: delta-aminolevulinic acid dehydratase, ferrochelatase, and coproporphyrinogen oxidase, but the major effectsareon ALAD activity. The δ-aminolevulinic acid dehydratase is the second enzyme of the heme pathway. This enzyme catalyzes the condensation of two molecules of δ-aminolevulinic acid (ALA) to form the monopyrrole porphobilinogen (PBG) [10]. In subsequent steeps, PBG is assembled into tetrapyrrole molecules, which constitute the prosthetic groups of hemoglobin [11]. Lead inhibition of ALAD activity results in accumulation of δ-aminolevulinic acid. ALA has been associated with oxidative damage by causing formation of reactive oxygen species (ROS), such as superoxide, hydroxyl radical, and hydrogen peroxide [12,13,14].

Negative correlations between blood lead concentration and ALAD activity have been reported, even at low levels of lead in blood [9,15,16]. On the other hand, positive correlations have been found between ALAD activity and malondialdehyde (MDA) levels [16]. Thus, ALAD activity is thought to be a sensitive indicator of early effect of lead as well as a biomarker of oxidative stress in the lead-exposed hematological system [17]. Blood lead has been considered a reliable indicator for the evaluation of lead exposure, whereas inhibition of ALAD activity has been considered one of the primary detectable parameters of lead poisoning [2].

Activity of ALAD is easily assayable in samples of peripheral blood. This enzyme has a high sensitivity to divalent lead ions, so it can be used as an indirect biomarker to estimate exposure to lead in humans [18]. ALAD activity test is considered appropriate for screening purposes, due to the progressive inactivation of this enzyme by lead over a range corresponding to subclinical intoxication [19]. In addition, ALAD activity is more sensitive than ALA in urine to evaluate the amount of circulating lead [9,20].

Previous epidemiological studies on the association between blood lead levels (BLLs) and ALAD activity showed divergent views. Studies reporting high levels of lead in blood revealed significant negative correlations between blood lead concentrations and ALAD activity [12,21,22]. However, some authors have demonstrated that ALAD inhibition occurs at levels of lead in blood around 5 µg/dL [15,16,23]. Most studies regarding the association between BLLs and ALAD activity have been conducted in occupationally exposed people and in children. Nevertheless, no significant variation of enzymatic ALAD activity has been reported in children at mean blood lead of 2.58 ± 0.30 µg/dL [13]. 

In a previous study, conducted by our research group, blood lead levels and some risk factors for lead exposure in pregnant women were determined, but ALAD activity was not evaluated [24]. The present cross-sectional study was designed to investigate the association between BLLs and ALAD activity in pregnant women from Durango, Mexico.

## 2. Materials and Methods

### 2.1. Subjects

This cross-sectional study was carried out between January 2014 and June 2016. The study subjects consisted of 633 clinically healthy pregnant women who received prenatal health care by the Secretariat of Health, State of Durango, Mexico. All pregnant women presented for prenatal care in health centers were asked to participate in the study. Those who accepted gave their written informed consent before being enrolled. Patients with renal failure, infectious disease or multifetal pregnancy were excluded. Participants were informed of the aims of the investigation and received information on ways to minimize their lead exposure. Each subject answered a questionnaire that contained sociodemographic data and information on reproductive history and sources of lead exposure. The study was conducted in accordance with the Declaration of Helsinki, and the research protocol was approved by the Ethical Committee of Durango General Hospital (approval number: 366/013). 

### 2.2. Sample Collection

For determination of ALAD activity, a venous blood sample was drawn for each patient and collected in vacutainer tubes using sodium heparin as an anticoagulant. A second sample was collected in lead-free vacutainer tubes containing ethylenediaminetetraacetic acid (EDTA), and separated in two portions; one for hematological analysis, and the remaining aliquot for lead level determination. Blood samples were collected before fasting. After collection, blood samples were transported in ice boxes to the Clinical Analysis Laboratory, Scientific Research Institute, Juarez University of the State of Durango. Samples were stored and transported in a lead-free environment to avoid any contamination, handled by trained personnel and kept in reserve at 4 °C.

### 2.3. Measurement of ALAD Activity

Enzyme activitywas assayed spectrophotometrically by the standardized European method [25]. The enzyme was incubated with excess δ-aminolevulinic acid at 37 °C. The porphobilinogen which was formed in 1 h was mixed with modified Ehrlich reagent. The color developed was measured spectrophotometrically at 555 nm against a blank. Results were expressed as δ-aminolevulinic acid, μmol/min per liter erythrocytes (U/L). The activity was determined no later than 10 h after the sample collection. 

### 2.4. Hematological Analysis

Hematological parameters were determined using an automated hematology analyzer (Abbott CELL-DIN 1400), at the Clinical Analysis Laboratory, Scientific Research Institute, Juarez Universityof the State of Durango. Red blood cells count (RBC), hemoglobin (Hb), hematocrit, meancorpuscular volume (MCV), mean corpuscular hemoglobin, and meancorpuscular hemoglobin concentration were determined. The hematocrit value was used for the calculation of the enzyme activity. Only hemoglobin value was presented in the results because of the possible relationship between hemoglobin and blood lead levels.

### 2.5. Determination of Lead in Blood

Blood samples were transferred to the Laboratory of Environmental Toxicology, Faculty of Medicine, Juarez University of the State of Durango, Gomez Palacio Campus. This laboratory participates in the Wisconsin State Laboratory Program of Hygiene proficiency testing (WSLPHT). Blood lead was measured using a graphite furnace atomic absorption spectrometer Perkin-Elmer AAnalyst 800 with Zeeman-effect background correction. Duplicates of blood samples were analyzed according to Miller et al. [26]. Lead in bovine blood from the National Institute of Standard and Technology (NIST) was used as standard reference material. Each sample duplicate was analyzed twice and those with variation coefficient above 5% were reanalyzed.

### 2.6. Statistical Analysis

The sociodemographic and reproductive characteristics were shown as mean ± standard deviation. The study population was divided into two groups: those with BLLs < 5 µg/dL and those with BLLs ≥ 5 µg/dL, and Student’s *t*-test was used to estimate differences between groups. To reduce the influence of extreme values on the statistical analysis, blood lead levels were analyzed by quartiles. One-way ANOVA was applied to compare the means between quartiles and the post-hoc comparisons were done using Tukey’s test. Pearson correlation analysis was carried out to evaluate the relationship of blood lead concentration with hemoglobin and ALAD activity in all groups. Multiple linear regression was performed to evaluate the association of ALAD activity with BLLs. Statistical analysis was carried out using Statistical Package for the Social Sciences (SPSS Inc., Chicago, IL, USA) software for Windows, version 15.0. A value of *p* < 0.05 was considered statistically significant.

## 3. Results

Table 1 summarizes the main characteristics, blood lead levels, and ALAD activity of women enrolled in this study. The mean age, education, gestational age, body mass index and hemoglobin of the studied population were 22.85 years, 10.04 years, 13.44 weeks, 25.61 kg/m^2^ and 13.00 g/dL, respectively. The mean income per capita accounted 99.55 United States Dollars (USD) per month (1 USD = 17.0 Mexican pesos). The mean level of blood lead was 2.09 ± 2.34 µg/dL; and the mean ALAD activity was 57.59 ± 21.12 U/L. 

Table 2 shows some characteristics for women with lead levels <5 µg/dL, and for women with lead levels ≥5 µg/dL. No significant differences between the groups were observed in age, education, gestational age, body mass index, monthly income per person and hemoglobin. However, ALAD activity was significantly lower in women with lead levels ≥5 µg/dL (*p* = 0.002).

Table 3 shows sociodemographic variables, hemoglobin and ALAD activity by quartiles of blood lead. A significant variation of ALAD activity was observed (*p* < 0.001). According to the Tukey test, women in the first quartile had the lowest ALAD activity. On the other hand, enzyme activity decreased between the third and the fourth quartiles. On the basis of these results, Pearson correlation was performed to determine the relation of blood lead concentration with hemoglobin and ALAD activity by quartiles of BLLs (Table 4). The correlation of BLLs with hemoglobin was not statistically significant. However, significant negative correlation between BLLs and ALAD activity was observed in the fourth quartile (r = −0.413; *p* < 0.01).

**Figure 1:**
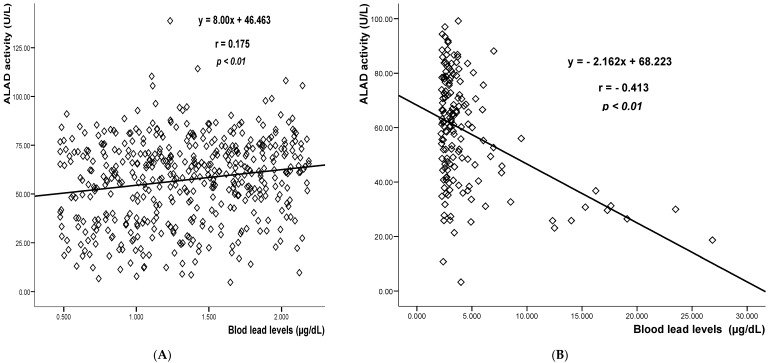
Linear regression between blood lead levels and δ-ALAD activity for women with BLLs < 2.2 µg/dL (**A**); and for thus with BLLs ≥ 2.2 µg/dL (**B**). The linear equation, correlation coefficient and *p* value are shown in the plot.

Taking into account the lower limit of blood lead for the third quartile, linear regression analysis was performed to determine the strength of the relationship between BLLs and ALAD activity in women with blood lead concentrations lower 2.2 µg/dL, and in those with BLLs ≥ 2.2 µg/dL (Figure 1). No significant association was observed between ALAD activity and BLLs for women with BLLs < 2.2 µg/dL. However, the results demonstrated a significant negative correlation (r = −0.413; *p* < 0.01) for women with BLLs ≥ 2.2 µg/dL. 

To deepen the exploration of the relationship between blood lead concentration and ALAD activity in women with BLLs ≥ 2.2 µg/dL, multiple linear regression was applied (Table 5). Blood lead levels were inversely associated with ALAD activity (*p* < 0.001). However, no significant associations were found for age, educational level, gestational age, body mass index and hemoglobin. The model represents 21.9% of the predictive capability.

## 4. Discussion

The mean blood lead concentration of 2.09 ± 2.34 µg/dL reported here is lower than those observed in other studies carried out in Mexican population. In Mexico City, Borja-Aburto found blood lead concentrations of 12.03 µg/dL in pregnant women who suffered spontaneous abortion and 10.09 µg/dL in a control group [4]. Another study of blood lead levels in pregnant women from Mexico City reported a mean blood lead concentration of 6.24 g/dL [27]. In a previous study carried out by our research group in pregnant women from Durango, Mexico, a mean blood lead level of 2.79 ± 2.14 µg/dL was observed, and 26 women (8.7%) had BLLs above the CDC recommended level of 5 µg/dL [24]. In the present research, also 26 women had levels of lead in blood above 5 µg/dL, but they represent 4.1% of the studied population.

Some authors have suggested that lead intoxication is characterized by high blood lead concentration and low ALAD activity [27,28]. For that reason, some researchers have recommended use of ALAD inhibition as an indicator of lead intoxication [12,21,29]. In our study, ALAD activity was significantly lower in women with BLLs ≥ 5 µg/dL compared with those with BLLs below 5 µg/dL. This finding is in an agreement with earlier published data. Similar results were observed in urban male adolescents from Lucknow, India [12], in children with neurological diseases from India [16], in lead workers from Taiwan [29], and in children from Southern Brazil [22]. 

Chiu et al. reported an inverse association between blood lead and ALAD activity when they compared lead workers from Taiwan with a control group (blood lead levels 19.5 ± 14.7 µg/dL and 2.9 ± 1.9 µg/dL, respectively) [29]. They concluded that the possible threshold value of blood lead for ALAD activity is around 10 µg/dL, and thus, ALAD activity may be usedas a biomarker for evaluation of lead toxicity in humans. Similar results were reported by Fecsa et al.; who analyzed lead dose-dependent effects for 18 lead exposed individuals and 12 normal volunteers [21]. Jasim et al. also reported a decrease of ALAD activity in battery manufacturing factory workers compared to non-exposed group; furthermore, this decrease became even more evident with increased duration of exposure [28]. The levels of lead in blood were 13.15 µg/dL in the control group, and more than 34.3 µg/dL in the exposed workers, respectively. In India, children residing in urban zones showed a negative correlation (*p* < 0.001) between blood lead levels (mean 11.8 ± 11.96 µg/dL) and ALAD activity [30].

Recent findings have suggested that ALAD inhibition may occur at low levels of lead in blood. Ahamed et al. reported a significant negative correlation between blood lead levels and ALAD activity in children with blood lead concentration lower than 10 µg/dL [15]. Moreover, Sakai and Morita considered that the threshold value of blood lead for ALAD inhibition is around 5 µg/dL [23]. Nevertheless, Martínez et al. did not find inhibition of enzymatic ALAD activity in children from Argentina, with mean blood lead of 2.58 ± 0.30 µg/dL [13]. 

Blood lead levels in our study were lower than in some prior studies on blood lead and ALAD activity [12,13,15,22,23,29,30]. Nevertheless, we observed a significant association between blood lead and ALAD activity at blood lead levels of 2.2 µg/dL, well below the CDC recommended level of 5 µg/dL for children and pregnant women [31]. To our knowledge, a similar result has not yet been reported in the literature.

It is well established that ALAD inhibition results in an increase of δ-ALA levels in blood, which can intensify oxidative stress and release iron from proteins such as ferritin [32]. For that reason, some authors have considered that decrease in ALAD activity has the potential to be used as an indicator of oxidative stress [32,33,34]. On the other hand, pregnancy is a condition that increases susceptibility to oxidative stress because of the mitochondria-rich placenta. During pregnancy, lipid peroxidation increases due to mitochondrial activity and hormone synthesis in placenta. Iron, which is abundant in the placenta, is important in the production of free radicals, and subjects the fetus to oxidative stress [35].

Importantly, our results also show that a small percent of pregnant women have blood lead concentrations above 5 µg/dL. Similar results were reported in a previous study carried out in Durango, Mexico [36]. A study conducted in Argentina, Mexico and Uruguay estimated 316,703 individuals in these countries are at risk of lead exposure, approximately 0.19% of the total population of all three countries. Of this population, 80,021 were women at childbearing age [37].

Researchers have documented that women with BLLs between 5–10 µg/dL have more probability of having a miscarriage compared to those with BLLs below 5 µg/dL [4]. It is thus necessary to identify and reduce the sources of exposure for these women. Recent research suggested a low threshold for the effect of maternal blood lead on birth outcomes, and recommended that exposure to lead during pregnancy should be kept as low as possible to minimize adverse outcomes [38]. Therefore, the growing evidence regarding the association between low levels of lead in blood and adverse pregnancy outcomes should be taken into account in the development of prevention politics. 

We recognized some limitations in our study. In Figure 1 samples with blood lead between 5 and 10 µg/dL show quite a dispersion, but even in this segment the correlation is negative. In contrast, samples with blood lead below 2.2 µg/dL showed a slight increase of ALAD activity. It is well established that ALAD activity is specifically inhibited by lead at concentrations between 5 and 50 µg/dL [9]. In spite of this, significant correlations were observed only in the fourth quartile (BLL >2.19 µg/dL). In the other hand, we did not evaluate some biomarkers of oxidative stress that may be associated with blood lead [39], which could have resulted in uncontrolled confounding. Alcohol consumption may affect ALAD activity, but it was not considered because only a few women recognized they had this habit. Nonetheless, to our knowledge, this is the first study which has analyzed the relationship between blood lead levels and ALAD activity in Mexican pregnant women. Moreover, in the revised literature, there is no such data evaluating the effect of lead exposure on enzymatic ALAD activity in pregnant women, who constitute one of the most vulnerable sections of the population.

## 5. Conclusions

In summary, the results of our study suggest that even very low lead exposure may cause a decrease of ALAD activity, at least in pregnant women. We propose that ALAD inhibition may occur at very low levels of lead in blood due to lead exposure and pregnancy conditions. 

## Figures and Tables

**Table 1:** Main characteristics of the studied subjects (n = 633). ALAD: delta-aminolevulinic acid dehydratase.

Variables	Mean ± SD *	Range
Age (years)	22.85 ± 6.35	13–43
Education (years)	10.04 ± 2.67	0.0–21.0
Gestational age (weeks)	13.44 ± 4.86	3.0–28.0
Body mass index (kg/m^2^)	25.61 ± 5.25	16.0–54.4
Income per capita (USD ** per month)	99.55 ± 89.68	4.41–970.59
Hemoglobin, g/dL	13.00 ± 1.27	8.8–23.1
Blood lead levels, µg/dL	2.09 ± 2.34	0.48–26.85
ALAD activity, U/L	57.59 ± 21.12	3.28–138.81

Note: * SD = standard deviation; ** USD = United States Dollars.

**Table 2:** Main characteristics of women with blood lead levels <5 µg/dL and ≥5 µg/dL. BLL: blood lead levels.

Variables	BLLs < 5 µg/dL (n = 607)	BLLs ≥ 5 µg/dL (n = 26)	*p* *
	Mean ± SD	Mean ± SD	
Age (years)	22.87 ± 6.36	22.42 ± 6.13	0.728
Education (years)	10.06 ± 2.68	9.58 ± 2.52	0.372
Gestational age (weeks)	13.46 ± 4.85	12.95 ± 5.06	0.612
Body mass index (kg/m^2^)	25.53 ± 5.20	27.36 ± 5.92	0.082
Income per capita (USD per month)	99.76 ± 78.70	94.32 ± 69.27	0.776
Hemoglobin, g/dL	13.00 ± 1.28	13.00 ± 1.04	0.974
ALAD activity, U/L	58.13 ± 21.05	45.10 ± 19.22	0.002

Note: * *p*-value was calculated from Student’s *t*-test.

**Table 3:** Change in demographic characteristics, hemoglobin and ALAD activity by quartiles of blood lead levels.

Variables	First Quartile	Second Quartile	Third Quartile	Fourth Quartile	p *
n	160	158	158	157	
BLLs (µg/dL)	<1.09	1.09–1.61	1.62–2.19	>2.19	
Age, years	22.50 ± 6.84	23.60 ± 6.13	23.10 ± 6.08	23.20 ± 6.36	0.696
Education (years)	10.10 ± 2.70	10.18 ± 2.73	9.81 ± 2.60	10.05 ± 2.72	0.637
Gestational age (weeks)	13.69 ± 4.98	13.47 ± 4.73	13.71 ± 4.94	12.86 ± 4.79	0.375
Body mass index (kg/m^2^)	24.90 ± 5.31	25.92 ± 5.39	26.01 ± 5.27	25.58 ± 4.98	0.254
Income per capita (USD per month)	98.41 ± 76.47	96.10 ± 100.04	95.99 ± 71.45	108.07 ± 106.64	0.614
Hemoglobin (g/dL)	12.88 ± 1.13	12.93 ± 1.20	12.95 ± 1.04	13.23 ± 1.64	0.070
ALAD activity, U/L	51.51 ± 21.82	59.10 ± 22.18	61.02 ± 19.10	58.82 ± 20.14	0.000

Note: * *p*-value was calculated from one-way ANOVA.

**Table 4:** Pearson correlations of blood lead levels with hemoglobin and ALAD activity by quartiles of blood lead levels.

Quartile of BLLs	Hemoglobin	ALAD Activity
First	0.027	−0.013
Second	−0.042	−0.043
Third	0.076	0.116
Fourth	−0.087	−0.413 **
All subjects	0.017	−0.113 **

Note: ** = Statistically significant correlation (*p* < 0.01).

**Table 5:** Multiple linear regression model for ALAD activity in women with BLLs ≥ 2.2 µg/dL (n = 142).

Variable	Coefficient β	Standard Error	*p-*Value
Age, years	0.239	0.261	0.361
Educational level, years	0.689	0.578	0.235
Gestational age, weeks	0.202	0.339	0.553
Body mass index (kg/m^2^)	−0.443	0.338	0.192
Hemoglobin (g/dL)	1.841	0.958	0.057
Blood lead levels (µg/dL)	−1.961	0.404	<0.001

Note: R^2^ = 0.219.
